# HIV Burden Trends in Kyrgyzstan, 1990–2025: A National Analysis of GBD 2021 Estimates and Short‐Term Projections

**DOI:** 10.1002/hsr2.73279

**Published:** 2026-09-22

**Authors:** Shafee Ur Rehman

**Affiliations:** ^1^ Department of Medicine Ala‐Too International University Bishkek Kyrgyzstan

**Keywords:** DALYs, epidemiology, Global Burden of Disease, HIV, Kyrgyzstan

## Abstract

**Background:**

Kyrgyzstan has experienced sustained HIV transmission, yet comprehensive national assessments of long‐term trends and demographic inequalities remain limited. This study evaluated the national HIV burden and benchmarked Kyrgyzstan's progress against Middle‐SDI and global patterns.

**Methods:**

We obtained HIV prevalence, mortality, and disability‐adjusted life years (DALYs) for 1990–2021 from the Global Burden of Disease Study 2021. Age‐standardized prevalence (ASPR), death (ASDR), and DALY rates per 100,000 population were examined by age and sex. Estimated annual percentage changes (EAPCs) with 95% confidence intervals were calculated using log‐linear regression. Values for 2022–2025 were short‐term projections derived by extrapolating 2010–2021 trends and were not official GBD estimates. We obtained transmission‐mode information from national surveillance sources and analyzed it descriptively.

**Results:**

HIV prevalence increased throughout 1990–2021 and was projected to reach an ASPR of 22.8 per 100,000 in 2025. Mortality and DALY rates peaked around 2010 and subsequently declined, although improvements were slower than global benchmarks. Adults aged 20–49 years experienced the greatest burden, while males generally had higher mortality and DALY rates than females. Surveillance data indicated an increasing proportion of diagnoses attributed to heterosexual exposure; however, recent evidence suggests possible underreporting of injecting‐drug‐use and male‐to‐male sexual exposure.

**Conclusions:**

Kyrgyzstan has achieved partial reductions in fatal HIV burden, but increasing prevalence indicates continuing transmission and long‐term treatment needs. Expanded testing, sustained antiretroviral therapy, harm reduction, and differentiated services for key populations and their sexual partners remain essential.

## 1 Introduction

Human immunodeficiency virus (HIV) remains a major global public health challenge despite substantial advances in prevention, diagnostic testing, and antiretroviral therapy (ART) [1, 2]. Since the beginning of the epidemic, HIV has caused millions of deaths and contributed substantially to morbidity, disability, and premature mortality [3]. Although global HIV incidence and AIDS‐related mortality have declined since 2010, primarily because of expanded ART access, the number of people living with HIV has continued to increase because of improved survival and ongoing transmission [4, 5]. The Global Burden of Disease (GBD) Study provides a standardized framework for evaluating these patterns using prevalence, mortality, years lived with disability, years of life lost, and disability‐adjusted life years (DALYs) [6, 7]. Age‐standardized rates and estimated annual percentage changes (EAPCs) permit the assessment of temporal trends and comparison of disease burden across populations.

Progress in controlling HIV has varied considerably among regions [8, 9, 10]. Eastern Europe and Central Asia remain of particular concern because the region has not experienced the sustained reductions observed in many other settings. In 2023, approximately 2.1 million people were living with HIV in Eastern Europe and Central Asia, while annual new infections and AIDS‐related deaths had increased by approximately 20% and 34%, respectively, since 2010 [11, 12]. Only about 59% of people living with HIV knew their status, 50% were receiving ART, and 42% had achieved viral suppression. These regional patterns are influenced by injecting drug use, population mobility, labor migration, stigma, unequal access to healthcare, incomplete prevention coverage, and changing sexual networks [11, 12, 13]. Nevertheless, regional averages conceal substantial differences among countries, highlighting the importance of nationally focused analyses.

Kyrgyzstan has experienced sustained HIV transmission since the 1990s. UNAIDS estimated that approximately 13,000 people were living with HIV in the country in 2024 [3, 14, 15]. Available national indicators suggest that the epidemic remains concentrated among key populations and their sexual partners. Estimated population sizes include approximately 25,000 people who inject drugs, 16,900 men who have sex with men, and 13,000 sex workers. Among sex workers, approximately 80% had reportedly undergone HIV testing or were aware of their HIV status [15]. These estimates should be interpreted cautiously because stigma, criminalization, mobility, and incomplete disclosure may underestimate key‐population sizes and misclassify transmission routes. A recent Kyrgyz study comparing registry classifications with confidential interviews found that injecting‐drug‐use exposure was reported more frequently during interviews than in routine registration. In contrast, heterosexual exposure was recorded more frequently in the registry [16]. Thus, the increasing proportion of cases classified as heterosexual transmission may reflect both genuine expansion into sexual and marital networks and incomplete disclosure of stigmatized exposures.

The national HIV response has expanded ART availability and increased domestic financing. Government funding reportedly covered approximately 90% of HIV treatment costs in 2022–2023, compared with 60% in 2021, and ART retention has improved substantially [17, 18]. Nevertheless, gaps remain in early diagnosis, prevention coverage, linkage to care, viral‐load monitoring, harm‐reduction services, and stigma‐free healthcare. These challenges may be particularly important for people who inject drugs, sex workers, men who have sex with men, migrants, prisoners, and the sexual partners of people from these populations. Routine surveillance provides essential information on diagnosed cases and treatment uptake. Still, it may not fully capture undiagnosed infections, long‐term demographic inequalities, or the combined fatal and non‐fatal burden of HIV.

Detailed national analyses of age‐ and sex‐specific prevalence, mortality, and DALYs in Kyrgyzstan remain limited. Accordingly, this study evaluated HIV burden using estimates from the GBD 2021 cycle for 1990–2021. Short‐term values for 2022–2025 were generated separately by extrapolating recent trends and should be interpreted as author‐derived projections rather than official GBD estimates. The study aimed to quantify temporal changes in prevalence, mortality, and DALYs; examine variations by age and sex; cautiously contextualize reported transmission patterns; and benchmark Kyrgyzstan against Middle‐SDI and global reference estimates. The findings are intended to support evidence‐based prevention, treatment planning, resource allocation, and national progress toward HIV‐control targets.

## 2 Methods

### 2.1 Study Design

This population‐based epidemiological study assessed the national burden of human immunodeficiency virus (HIV) infection in Kyrgyzstan. Historical analyses covered 1990–2021, corresponding to the Global Burden of Disease Study 2021 (GBD 2021) estimation cycle. A separate forecasting analysis extended the trends from 2022 to 2025. Accordingly, values reported for 1990–2021 represent GBD estimates, whereas values for 2022–2025 represent author‐derived short‐term projections and should not be interpreted as official GBD estimates. The study examined temporal changes in HIV prevalence, mortality, and disability‐adjusted life years (DALYs), with stratification by age and sex and descriptive contextualization of reported transmission routes.

### 2.2 Data Sources

We obtained country‐, year‐, age‐, and sex‐specific HIV estimates from the GBD 2021 Results Tool maintained by the Institute for Health Metrics and Evaluation. Extracted measures included prevalence, deaths, years of life lost (YLLs), years lived with disability (YLDs), and DALYs, expressed as both counts and rates per 100,000 population. We retained point estimates and corresponding 95% uncertainty intervals (UIs). GBD 2021 integrates information from national surveillance systems, vital registration records, disease registries, published epidemiological studies, and international health databases through standardized data‐processing and modeling procedures. We used UNAIDS country reports, national HIV surveillance summaries, and recent empirical studies to contextualize ART coverage, key‐population estimates, and reported transmission patterns. These contextual data were analyzed separately and were not statistically combined with GBD estimates.

### 2.3 Case Definition

HIV and AIDS outcomes were defined according to the GBD 2021 cause hierarchy and the corresponding International Classification of Diseases codes. The case definition included both symptomatic and asymptomatic HIV infection, as well as AIDS‐related conditions and deaths attributable to HIV. Transmission route is not a standard GBD cause measure. Consequently, information on heterosexual exposure, injecting drug use, male‐to‐male sexual exposure, mother‐to‐child transmission, and other routes was derived from national and UNAIDS surveillance sources and analyzed descriptively. These registered categories were not treated as definitive biological attribution because stigmatized exposures may be underreported or misclassified.

### 2.4 Measures of Disease Burden

The primary outcomes were HIV prevalence, mortality, and DALYs. Prevalence was defined as the estimated number of people living with HIV during a specified year. Mortality represented deaths attributable to HIV and AIDS‐related causes. DALYs were calculated as the sum of YLLs due to premature mortality and YLDs associated with non‐fatal health loss. YLLs were estimated by multiplying the number of deaths at each age by the remaining standard life expectancy. In contrast, YLDs were estimated from the prevalence and severity of HIV‐related health states using GBD disability weights. Age‐standardized prevalence rates (ASPRs), age‐standardized death rates (ASDRs), and age‐standardized DALY rates were expressed per 100,000 population and standardized using the GBD world standard population.

### 2.5 GBD Estimation and Uncertainty Framework

GBD 2021 applies standardized modeling procedures to harmonize heterogeneous epidemiological and mortality data across countries and years. Cause‐specific mortality estimates are reconciled with independently estimated all‐cause mortality, whereas non‐fatal HIV outcomes are estimated using disease‐modeling and Bayesian meta‐regression approaches. DALYs are calculated by combining modeled YLLs and YLDs. The GBD uncertainty framework propagates uncertainty arising from sampling variability, incomplete data, measurement error, data correction, and model specification across repeated model draws. We reported the 2.5th and 97.5th percentiles of the ordered draws as the lower and upper limits of the 95% UI. We did not interpret overlapping UIs as a formal statistical test of equality.

### 2.6 Trend Analysis

Temporal trends in age‐standardized HIV prevalence, mortality, and disability‐adjusted life‐year rates were evaluated using the estimated annual percentage change (EAPC). For each outcome, the natural logarithm of the annual age‐standardized rate was regressed on calendar year using the following log‐linear model:

$$\ln(R_{y})=\alpha +\beta y+\varepsilon_{y}$$

where Rᵧ denotes the age‐standardized rate in year y, α represents the intercept, β is the regression coefficient describing the annual change in the logarithm of the rate, and εᵧ is the random error term. The EAPC was derived from β as follows:

EAPC=100×[exp(β)−1]

The corresponding 95% confidence interval (CI) was calculated as:

95%CI=100×{exp[β±1.96×SE(β)]−1}

where SE(β) denotes the standard error of the regression coefficient; a temporal trend was classified as increasing when both the EAPC estimate and the lower bound of its 95% CI were greater than zero. It was classified as decreasing when both the EAPC estimate and the upper bound of the 95% CI were below zero. When the 95% CI included zero, the trend was considered statistically stable. EAPCs were estimated for the full GBD 2021 observation period (1990–2021) and separately for 2010–2021 to characterize recent changes following the observed peak in HIV mortality and DALY rates around 2010. All slope tests were two‐sided, and *p* < 0.05 was considered statistically significant.

### 2.7 Short‐Term Projections to 2025

Values for 2022–2025 were projected by extrapolating the log‐linear trends observed during 2010–2021. For each outcome, the fitted model generated annual point predictions and 95% prediction intervals. This continuation‐of‐recent‐trend approach assumed that the average post‐2010 rate of change would remain approximately constant through 2025. The projections did not account for unforeseen changes in migration, HIV testing, ART coverage, treatment adherence, prevention funding, surveillance practices, or national policy. Therefore, the projected estimates represent scenario‐based forecasts rather than official GBD 2025 estimates. Sensitivity analyses compared projections generated from the 2005–2021 and 2010–2021 fitting periods to evaluate the influence of the selected forecasting window.

### 2.8 Subgroup Analyses

We conducted age‐specific analyses across GBD age groups and summarized them into clinically interpretable life‐course categories where appropriate. Sex‐specific analyses compared males and females by prevalence, mortality, and DALYs. We calculated male‐to‐female rate ratios to quantify sex inequalities. Transmission patterns were described using national surveillance classifications, including heterosexual exposure, injecting drug use, male‐to‐male sexual exposure, mother‐to‐child transmission, and other or unknown routes. Because confidential behavioral studies have identified possible misclassification of registered transmission categories in Kyrgyzstan, the transmission‐mode analysis was interpreted cautiously. It was not used to infer a definitive transition to a generalized epidemic.

### 2.9 Comparative Analysis

We compared Kyrgyzstan's ASPR, ASDR, and age‐standardized DALY rates with the corresponding GBD 2021 global and Middle‐SDI aggregate estimates. We used identical outcome definitions, calendar years, rate denominators, and age‐standardization procedures for all comparisons. We calculated Kyrgyzstan‐to‐reference rate ratios where the necessary estimates were available. These comparisons were intended to benchmark national progress and identify differences in epidemic trajectories; they were not designed as a multicountry causal analysis.

### 2.10 Statistical Analysis

Data management, statistical analyses, and visualizations were performed using R version 4.4.0. We reported continuous estimates with 95% UIs, while regression‐derived EAPCs and comparative statistics were presented with 95% CIs. All statistical tests were two‐sided, and *p* < 0.05 was considered statistically significant. We evaluated model assumptions by inspecting residuals, fitted‐versus‐observed plots, and influential observations. Figures differentiated historical GBD estimates from projected values using separate line types or shaded forecast regions. The analytical code, extracted dataset, GBD query specifications, and forecasting parameters should be provided as supporting materials to support reproducibility.

## 3 Results

### 3.1 Overall HIV Burden in Kyrgyzstan, 1990–2025

**Figure 1:**
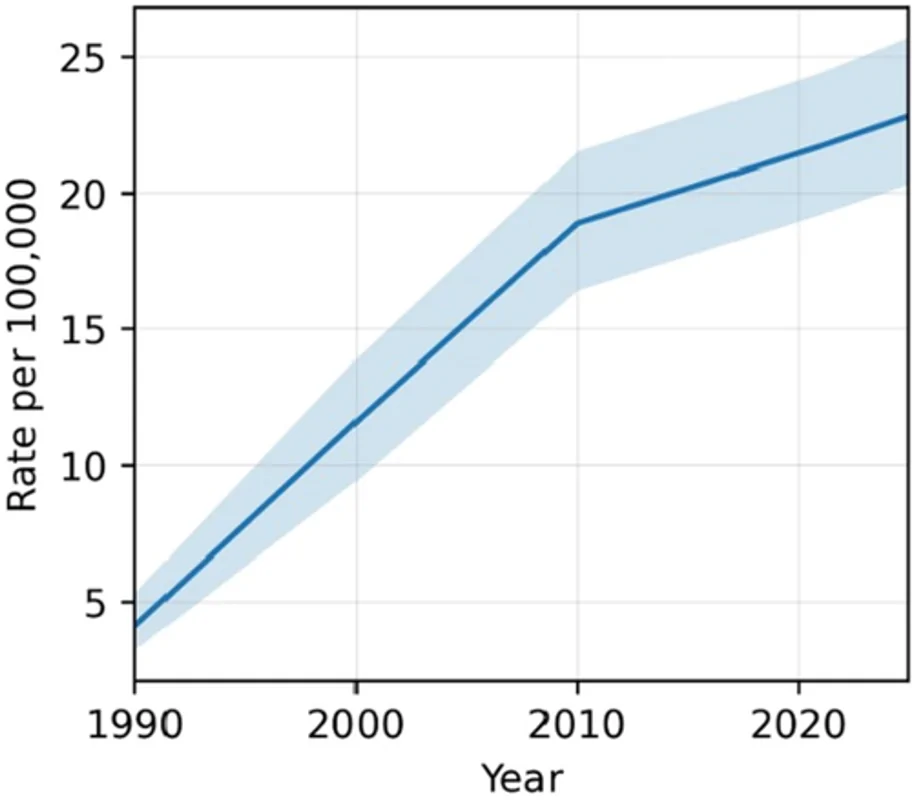
Age‐standardized prevalence rate (ASPR) of HIV per 100,000 population in Kyrgyzstan from 1990 to 2025. Solid lines represent point estimates, and shaded areas indicate 95% uncertainty intervals. Trends were evaluated using estimated annual percentage change (EAPC). Rates were age‐standardized using the Global Burden of Disease standard population.

GBD 2021 estimates demonstrated a substantial increase in HIV prevalence in Kyrgyzstan between 1990 and 2021. The estimated number of people living with HIV increased from approximately 1,020 in 1990 to 3,450 in 2000, 7,980 in 2010, and 12,430 in 2021. Under the continuation‐of‐trend projection model, this number was projected to reach approximately 15,180 in 2025 (Table 1). The age‐standardized prevalence rate (ASPR) increased from 4.1 per 100,000 population in 1990 to 11.6 in 2000, 18.9 in 2010, and 21.7 in 2021. Between 2010 and 2021, the ASPR increased at an average annual rate of 1.26%. Continuation of this trend produced a projected ASPR of 22.8 per 100,000 in 2025 (Figure 1). These results indicate that the rate of increase slowed after 2010 but did not reverse. The simultaneous increase in prevalence and reduction in mortality is compatible with both continuing transmission and improved survival among people receiving antiretroviral therapy (ART). However, these mechanisms could not be evaluated directly using the aggregated GBD data.

**Table 1:** HIV burden in Kyrgyzstan, 1990–2021, with short‐term projections to 2025.

Indicator	1990	2000	2010	2021	2025 projection^a^	Annual change, 2010–2021
People living with HIV, *n*	~1,020	~3,450	~7,980	~12,430	~15,180	+4.11%
ASPR per 100,000	4.1	11.6	18.9	21.7	22.8	+1.26%
HIV‐related deaths, *n*	~120	~460	~680	~520	~470	−2.40%
ASDR per 100,000	0.6	2.4	3.1	2.2	1.9	−3.07%
Total DALYs, *n*	~9,800	~38,400	~56,900	~49,200	~45,600	−1.31%
Age‐standardized DALY rate per 100,000	42.5	148.2	201.6	172.4	158.3	−1.41%
Male proportion of DALYs	—	—	—	Approximately 60%–65%	Approximately 60%–65%	—
Female proportion of DALYs	—	—	—	Approximately 35%–40%	Approximately 35%–40%	—
Highest‐burden age group	—	—	—	20–49 years	20–49 years	—

^a^ GBD 2021 estimates cover 1990–2021. Values for 2022–2025 are author‐derived short‐term projections and are not official GBD estimates. Annual changes were calculated from the 2010 and 2021 endpoints as 100×[(R2010R2021)111 − 1]. They represent annualized rates of change rather than regression‐derived EAPCs with 95% confidence intervals. ASPR, age‐standardized prevalence rate; ASDR, age‐standardized death rate; DALYs, disability‐adjusted life years.

### 3.2 Trends in HIV Mortality

**Figure 2:**
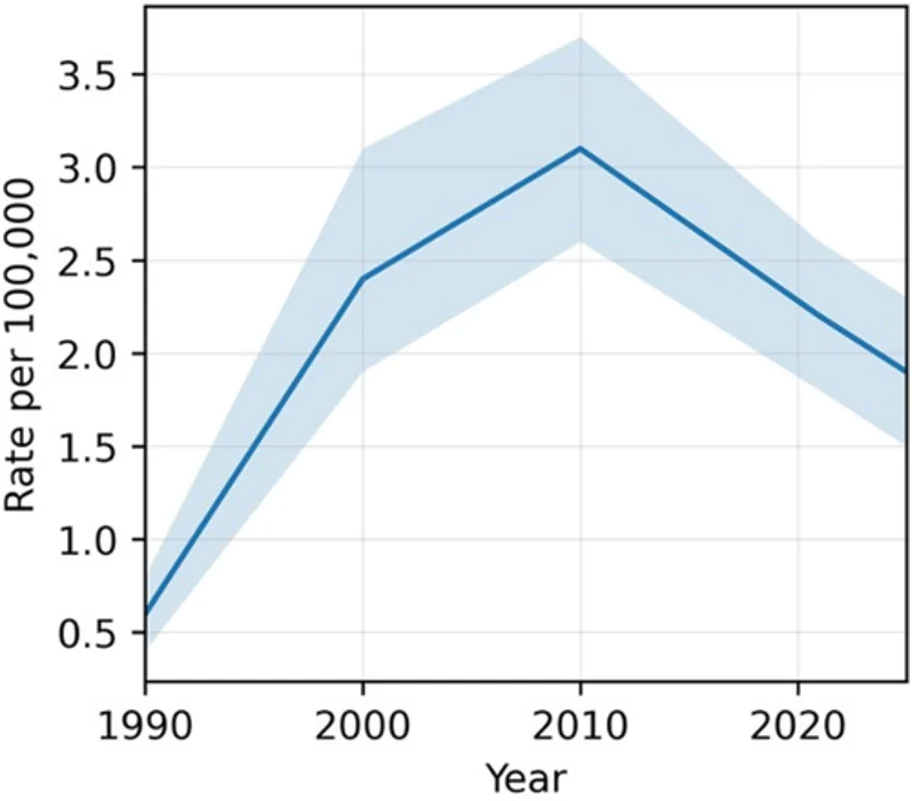
Age‐standardized death rate (ASDR) attributable to HIV per 100,000 population over the same period. Solid lines represent point estimates, and shaded areas indicate 95% uncertainty intervals. Trends were evaluated using estimated annual percentage change (EAPC). Rates were age‐standardized using the Global Burden of Disease standard population.

HIV‐related mortality increased during the earlier phase of the epidemic. The estimated number of HIV‐related deaths rose from approximately 120 in 1990 to 460 in 2000 and reached approximately 680 in 2010. It subsequently declined to approximately 520 in 2021, representing an average annual reduction of 2.40% during 2010–2021. The corresponding continuation‐of‐trend projection suggested approximately 470 deaths in 2025. The ASDR increased from 0.6 per 100,000 in 1990 to 2.4 in 2000 and peaked at 3.1 per 100,000 in 2010. It then declined to 2.2 per 100,000 in 2021, corresponding to an average annual reduction of 3.07% during 2010–2021. Under the projection model, the ASDR was expected to decline further to 1.9 per 100,000 in 2025 (Figure 2). Despite the post‐2010 reduction, the estimated 2021 and projected 2025 rates remained higher than the 1990 rate.

### 3.3 Trends in DALYs Attributable to HIV

**Figure 3:**
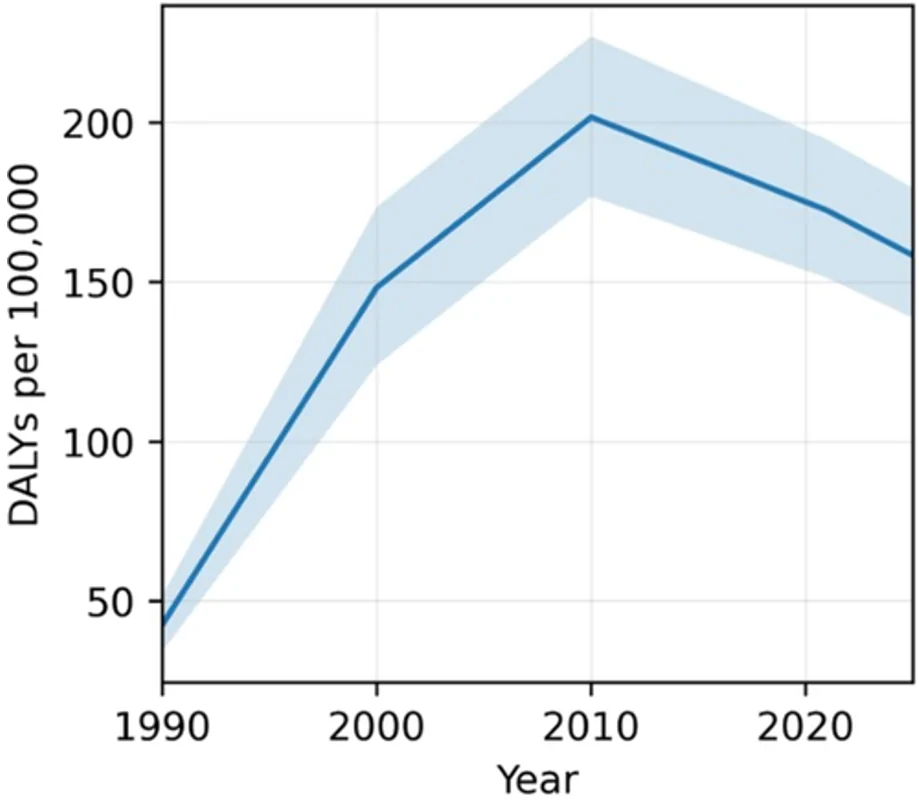
Age‐standardized disability‐adjusted life year (DALY) rate (ASR‐DALYs) per 100,000 population. Solid lines represent point estimates, and shaded areas indicate 95% uncertainty intervals. Trends were evaluated using estimated annual percentage change (EAPC). Rates were age‐standardized using the Global Burden of Disease standard population.

The total number of HIV‐attributable DALYs increased from approximately 9,800 in 1990 to 38,400 in 2000 and 56,900 in 2010. It subsequently declined to approximately 49,200 in 2021, representing an average annual reduction of 1.31% during 2010–2021. Continuing this trend would have produced a projected total of approximately 45,600 DALYs in 2025. The age‐standardized DALY rate followed a similar pattern. It increased from 42.5 per 100,000 in 1990 to 148.2 in 2000 and 201.6 in 2010 before declining to 172.4 per 100,000 in 2021. This decline corresponded to an average annual reduction of 1.41% from 2010 to 2021. The rate was projected to decrease further to 158.3 per 100,000 in 2025 (Figure 3). Nevertheless, the projected burden remained considerably higher than the corresponding 1990 level.

### 3.4 Age‐Specific Patterns

**Figure 4:**
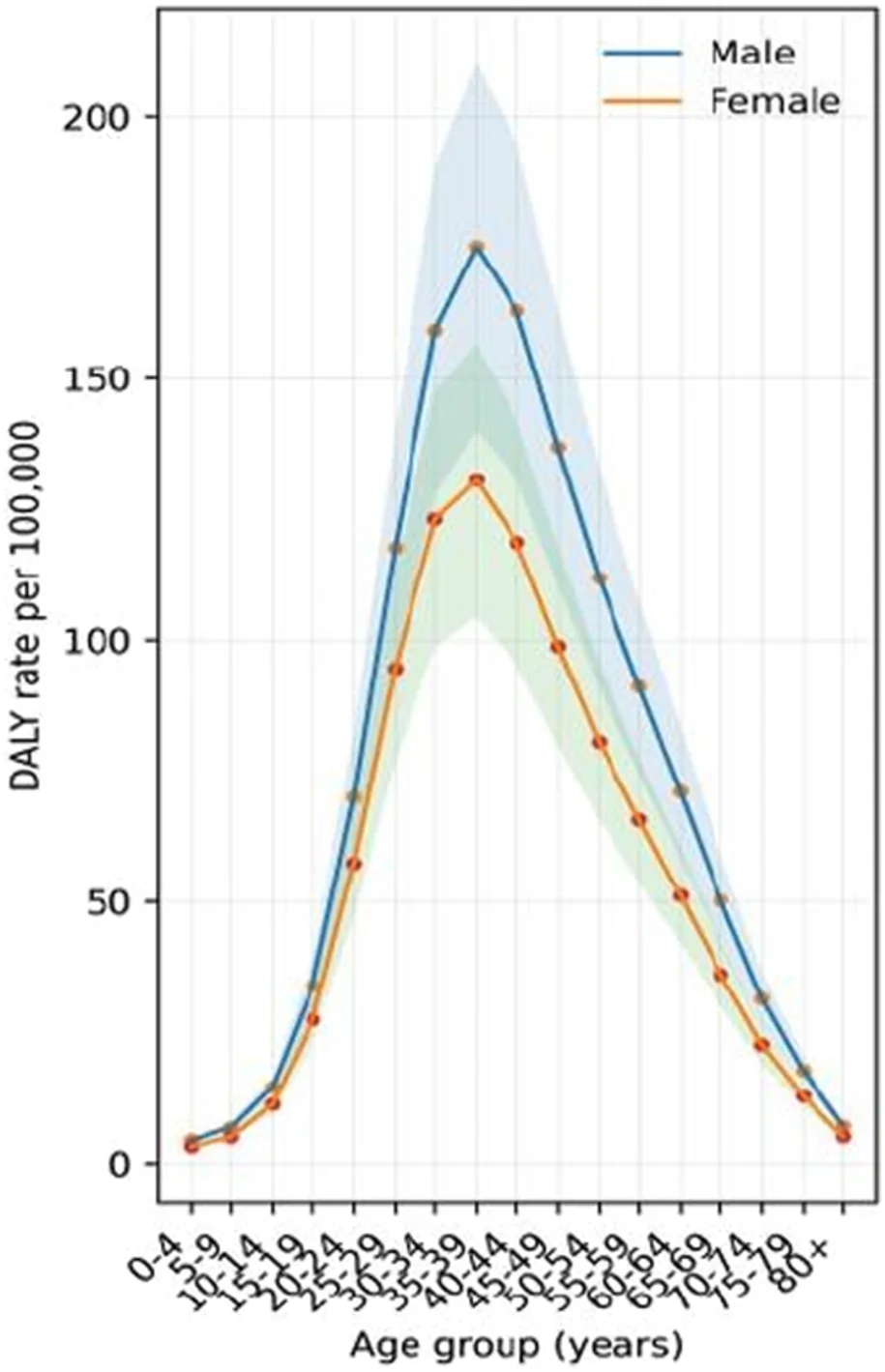
Age‐ and sex‐specific HIV DALY rates in Kyrgyzstan, 2025. Age‐ and sex‐specific DALY rates per 100,000 population attributable to HIV in Kyrgyzstan in 2025. DALY rates are shown across age groups for males and females. Shaded bands represent uncertainty intervals. The highest burden is observed among adults aged 20–49 years, with males experiencing consistently higher DALY rates than females across most age groups.

The distribution of HIV‐attributable DALYs varied substantially across age groups. Adults aged 20–49 years accounted for more than 65% of the total burden, with the highest rates occurring among adults aged approximately 30–44 years (Figure 4). DALY rates increased rapidly after adolescence, peaked during middle adulthood, and declined progressively in older age groups. Children and adolescents younger than 15 years accounted for a relatively small proportion of the total DALY burden. The persistence of pediatric HIV burden was compatible with continuing vertical transmission and survival among children living with HIV. However, the GBD estimates did not directly identify the transmission routes responsible for pediatric cases.

### 3.5 Sex‐Specific Differences

Males experienced a greater HIV‐related burden than females across most adult age groups. Approximately 60%–65% of total HIV‐attributable DALYs occurred among males, compared with approximately 35%–40% among females. The largest sex differences were observed after 35 years of age, when male mortality and DALY rates were consistently higher (Figure 4). Although prevalence was broadly comparable between the sexes, the mortality and DALY burden were higher among males. Possible explanations include differences in injecting‐drug‐use exposure, labor migration, delayed diagnosis, healthcare engagement, and treatment retention. These explanations were not tested directly and should therefore be interpreted as hypotheses rather than causal findings.

### 3.6 Comparison with Global and Middle‐SDI Benchmarks

**Figure 5:**
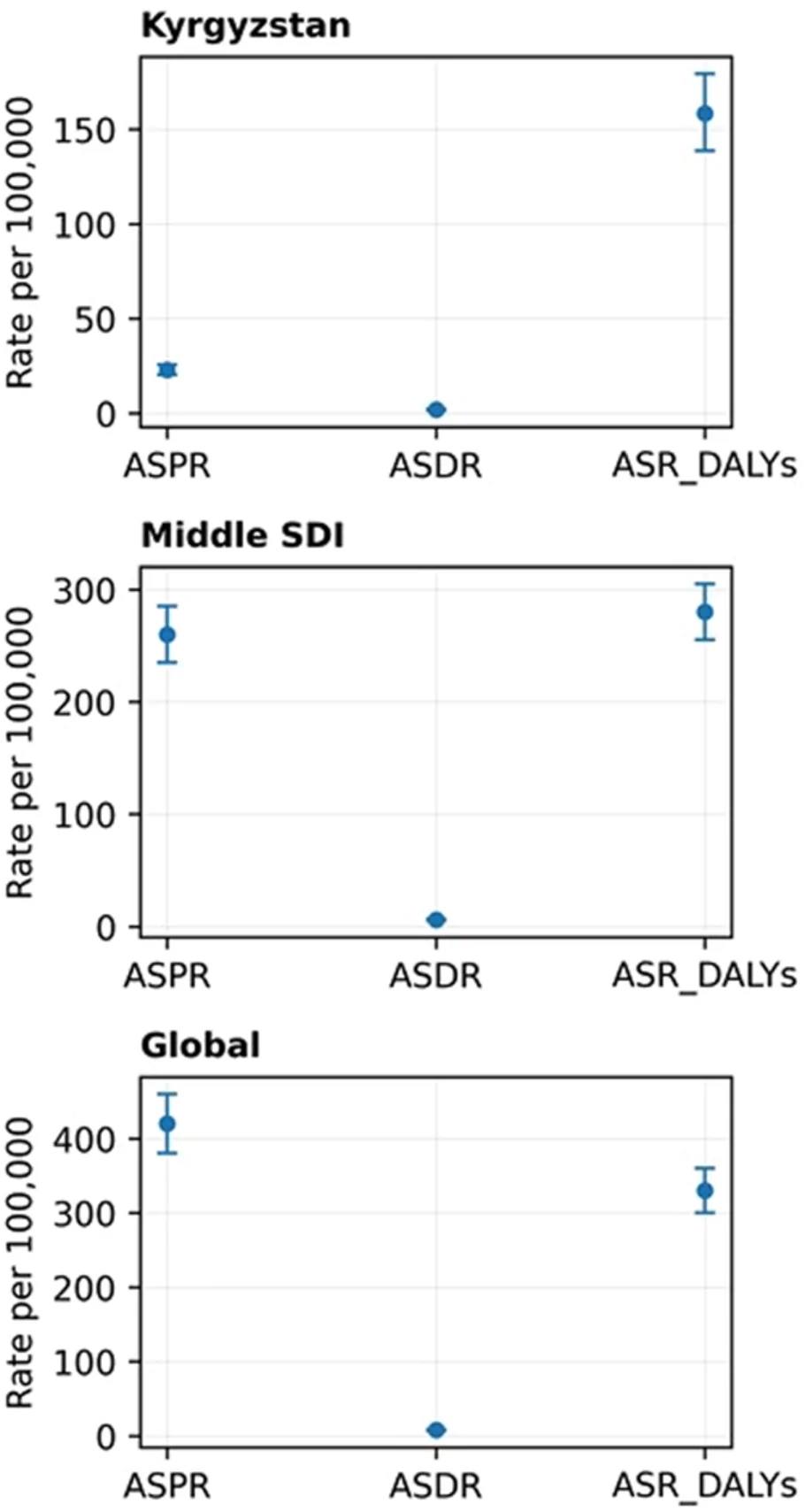
Comparison of HIV burden in Kyrgyzstan with Middle‐SDI and Global benchmarks, 2025. Comparison of age‐standardized HIV prevalence (ASPR), mortality (ASDR), and DALY (ASR‐DALYs) rates per 100,000 population in Kyrgyzstan, Middle–socio‐demographic index (SDI) countries, and global averages in 2025. Points indicate central estimates and error bars represent uncertainty intervals. Kyrgyzstan shows faster prevalence growth and slower declines in mortality and DALYs than global benchmarks.

In 2021, Kyrgyzstan had an ASPR of 21.7 per 100,000, an ASDR of 2.2 per 100,000, and an age‐standardized DALY rate of 172.4 per 100,000. The corresponding 2025 projections were 22.8, 1.9, and 158.3 per 100,000, respectively. Kyrgyzstan's absolute age‐standardized rates were lower than the corresponding Middle‐SDI and global aggregate rates presented in Figure 5. However, its temporal trajectory was less favorable because prevalence continued to increase, while post‐2010 reductions in mortality and DALYs were comparatively modest. During 2010–2021, Kyrgyzstan's ASPR increased by an average of 1.26% annually, whereas its ASDR and age‐standardized DALY rate declined by 3.07% and 1.41% annually, respectively. These comparisons indicate partial progress in reducing fatal HIV burden but continuing challenges in controlling prevalence.

### 3.7 Reported Transmission‐Mode Patterns

**Figure 6:**
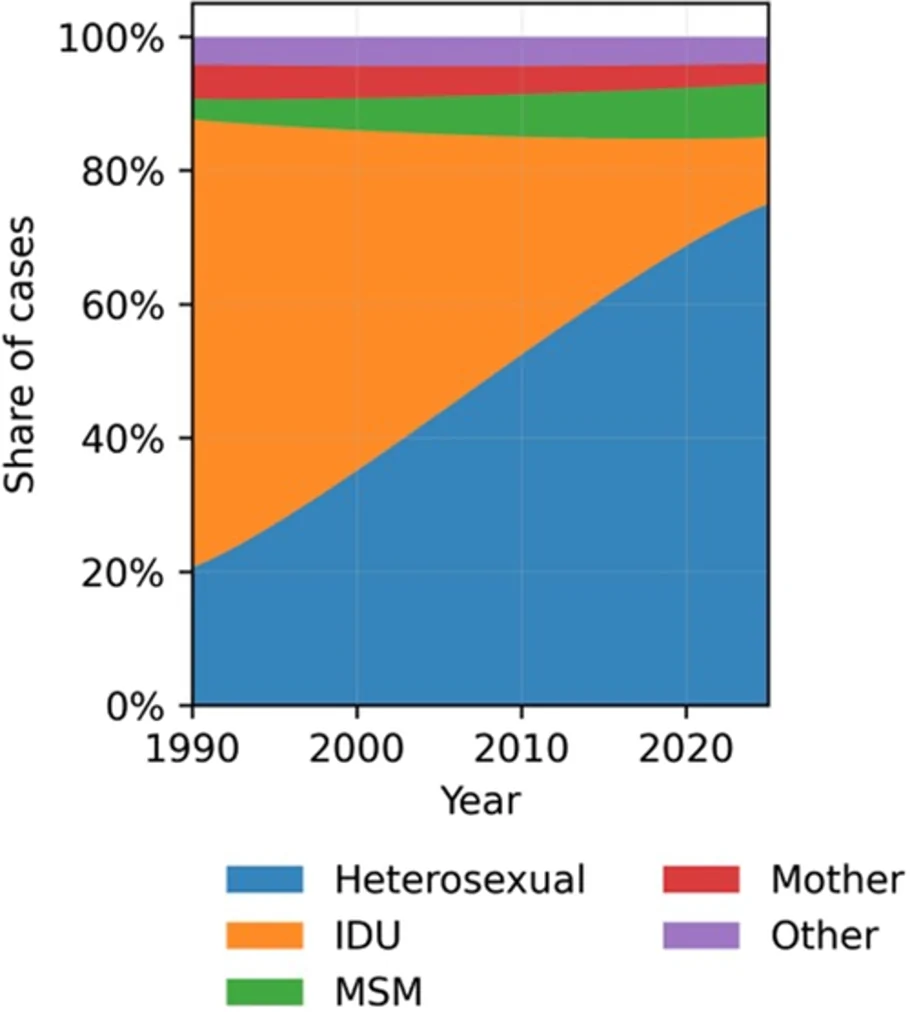
Distribution of HIV transmission modes in Kyrgyzstan, 1990–2025. Stacked area plot showing the proportional contribution of major HIV transmission routes in Kyrgyzstan from 1990 to 2025, including heterosexual transmission, injecting drug use (IDU), men who have sex with men (MSM), mother‐to‐child transmission, and other routes. The figure illustrates a transition from an IDU‐dominated epidemic in the early period to predominantly heterosexual transmission in recent years.

National surveillance data indicated a change in registered HIV exposure categories during the study period. Injecting‐drug‐use exposure represented the predominant registered category during the earlier epidemic, accounting for approximately 65% of reported cases in 1990. Its reported contribution subsequently declined and was projected at approximately 15% in 2025. Conversely, the proportion of cases registered as heterosexual exposure increased from approximately 20% in 1990 to approximately 75% in 2025 (Figure 6). Male‐to‐male sexual exposure represented a smaller but gradually increasing proportion of registered cases, while mother‐to‐child and other reported transmission categories remained comparatively stable. These findings should not be interpreted as definitive evidence of a complete transition to a generalized heterosexual epidemic. Recent confidential interview data from Kyrgyzstan suggest that injecting‐drug‐use and male‐to‐male sexual exposure may be underreported, while heterosexual exposure may be over‐assigned in routine surveillance. The apparent change may therefore reflect genuine expansion into sexual and marital networks combined with migration, behavioral changes, non‐disclosure, and exposure misclassification.

### 3.8 Summary of Principal Findings

**Figure 7:**
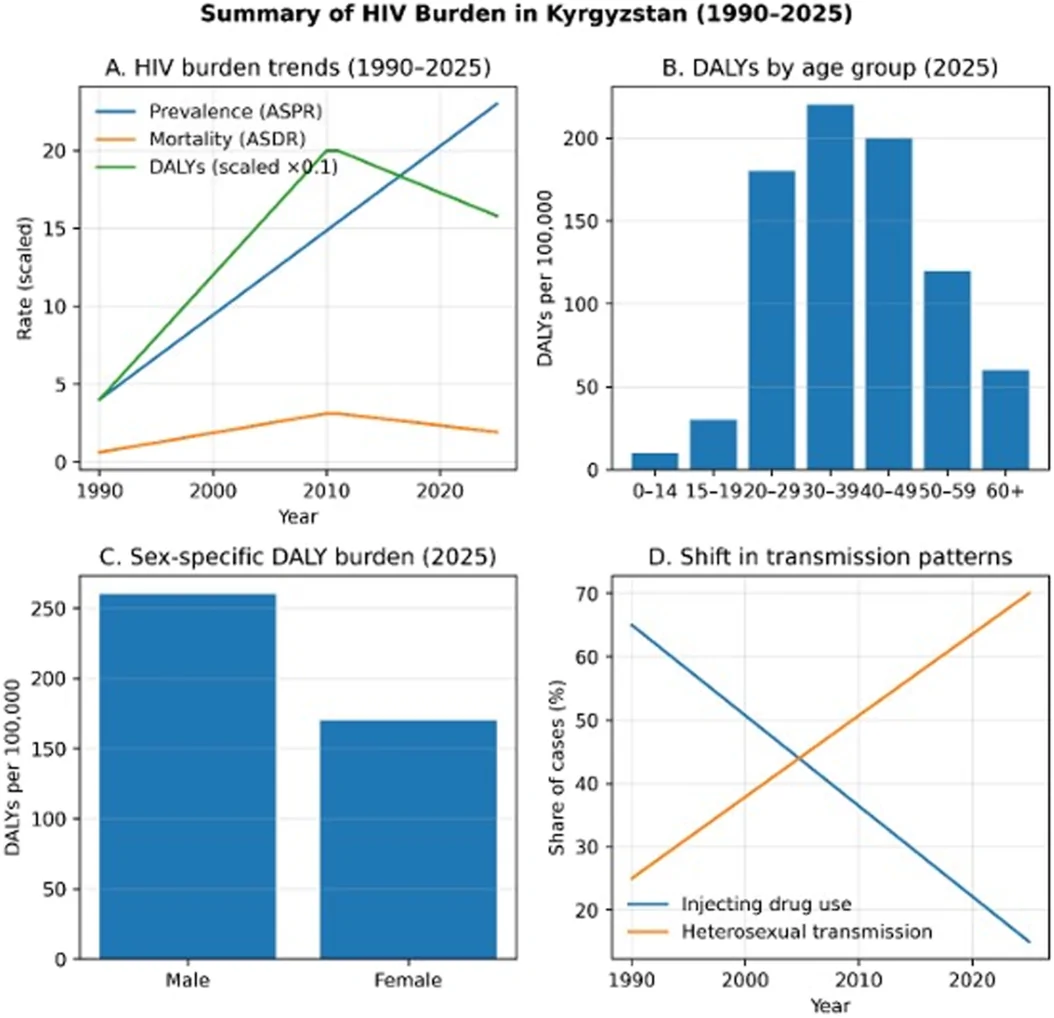
Summary of HIV burden patterns in Kyrgyzstan, 1990–2025. Multi‐panel summary figure illustrating key epidemiological findings. (A) Temporal trends in HIV prevalence, mortality, and DALYs. (B) Age‐specific DALY distribution in 2025, highlighting the highest burden among adults aged 20–49 years. (C) Sex‐specific DALY burden in 2025, showing higher rates among males. (D) Temporal shift in dominant HIV transmission modes from injecting drug use to heterosexual transmission.

Four principal findings emerged from the analysis. First, HIV prevalence increased substantially between 1990 and 2021, and this increase was projected to continue through 2025. Second, HIV‐related mortality and DALY rates peaked around 2010 and subsequently declined, with average annual reductions of 3.07% and 1.41%, respectively, during 2010–2021. Third, adults aged 20–49 years accounted for more than 65% of HIV‐attributable DALYs, while approximately 60%–65% of the total DALY burden occurred among males. Fourth, the proportion of cases registered as heterosexual exposure increased as injecting‐drug‐use exposure declined; however, this finding must be interpreted cautiously because surveillance classifications may be affected by non‐disclosure and misclassification (Figure 7).

## 4 Discussion

This study provides a national assessment of HIV burden in Kyrgyzstan using GBD 2021 estimates for 1990–2021 and author‐derived short‐term projections for 2022–2025. HIV prevalence increased substantially over the observed period, whereas mortality and DALY rates peaked around 2010 and subsequently declined. Between 2010 and 2021, the ASPR increased by an average of 1.26% annually, while the ASDR and age‐standardized DALY rate decreased by 3.07% and 1.41% annually, respectively. Continuation of these trends produced projected 2025 rates of 22.8 for prevalence, 1.9 for mortality, and 158.3 DALYs per 100,000 population. These projections represent a continuation‐of‐recent‐trend scenario and should not be interpreted as official GBD estimates.

The contrasting increase in prevalence and decline in fatal burden is compatible with improved survival following ART expansion alongside continuing HIV transmission. However, the ecological GBD data did not permit direct separation of the effects of ART, earlier diagnosis, incidence, or changes in population structure. Adults aged 20–49 years accounted for more than 65% of HIV‐attributable DALYs, and approximately 60–65% of the total DALY burden occurred among males. These findings identify economically and socially active adults, particularly men, as important populations for intensified testing, prevention, and sustained engagement in care [15, 16, 17].

### 4.1 Comparison With Regional and Global Trends

Kyrgyzstan's HIV trajectory should be interpreted within the broader epidemiological context of Eastern Europe and Central Asia. Unlike regions that have experienced sustained reductions in new infections and AIDS‐related mortality, Eastern Europe and Central Asia recorded increases in both indicators after 2010. UNAIDS estimated that the region experienced a 20% increase in new HIV infections and a 34% increase in AIDS‐related deaths between 2010 and 2023. Treatment‐cascade indicators also remained below international targets: approximately 59% of people living with HIV knew their status, 50% were receiving ART, and 42% had achieved viral suppression in 2023 [17, 18, 19].

Kyrgyzstan's absolute age‐standardized HIV rates were lower than some Middle‐SDI and global aggregate estimates; however, its temporal trajectory remained concerning because prevalence continued to increase and reductions in mortality and DALYs were comparatively modest. The post‐2010 decline in mortality may reflect improved ART availability, increasing domestic financing, and better retention in care. Nevertheless, continued prevalence growth suggests that reductions have not kept pace with treatment gains in transmission. These findings are consistent with recent Central Asian studies reporting persistent barriers related to delayed diagnosis, stigma, incomplete prevention coverage, drug resistance, and unequal access to services among key populations [19, 20, 21, 22]. International benchmarking therefore indicates partial progress in reducing fatal burden but insufficient progress toward controlling the national epidemic.

### 4.2 Interpretation of Changing Transmission Patterns

The increasing proportion of diagnoses registered as heterosexual exposure should not be interpreted as definitive evidence that a generalized heterosexual epidemic has replaced a previously concentrated injecting‐drug‐use epidemic. Routine surveillance categories describe reported or assigned exposure routes and may not always correspond to the underlying biological transmission network. A recent study from Kyrgyzstan comparing registry classifications with confidential interviews found that injecting‐drug‐use exposure was reported more frequently during interviews than in the national registry. In contrast, heterosexual exposure was recorded more frequently in routine surveillance [22, 23]. This finding suggests that stigmatized exposures, including injecting drug use and male‐to‐male sexual contact, may be underreported.

Migration may also contribute to the increasing number of infections identified through heterosexual networks. Kyrgyzstan has extensive internal mobility and international labor migration, particularly among working‐age men. Circular migration, prolonged separation from spouses, new sexual partnerships while away from home, and delayed testing after return may connect geographically separated sexual networks. Marital mobility can consequently increase risk among women who do not identify as members of a key population but whose partners have injecting‐drug‐use, male‐to‐male sexual, or commercial‐sex exposures. Limited disclosure within marriage and low perceived risk may further delay testing among spouses.

Behavioral changes may also influence the distribution of registered transmission categories. These include changing patterns of injecting drug use, inconsistent condom use, expansion of digital partner‐seeking, commercial or transactional sex, and increased sexual mixing between key populations and the wider population [24, 25]. Greater awareness of HIV may improve disclosure of some exposures, whereas stigma and fear of social or legal consequences may suppress disclosure of others. The apparent rise in heterosexual transmission therefore probably reflects overlapping effects of genuine sexual transmission, migration, marital and partner mobility, behavioral change, and exposure misclassification. Prevention strategies should address these interconnected networks rather than treating heterosexual populations and traditional key populations as separate epidemiological groups.

### 4.3 Policy and Public‐Health Implications

The findings have several implications for Kyrgyzstan's national HIV response. First, the continuing increase in prevalence supports expansion of voluntary and confidential testing, including provider‐initiated testing in primary care, community‐based testing, self‐testing, and partner‐notification services. Testing programs should prioritize adults aged 20–49 years while maintaining differentiated services for people who inject drugs, men who have sex with men, sex workers, prisoners, migrants, and their sexual partners. Second, the greater mortality and DALY burden among males indicates a need for male‐responsive services. HIV testing could be integrated into occupational health programs, migrant health services, harm‐reduction facilities, tuberculosis services, and other settings commonly accessed by working‐age men. Flexible clinic hours, rapid linkage to ART, peer navigation, and decentralized medication delivery may improve retention among mobile populations.

Third, the increasing number of cases registered as heterosexual exposure requires broader access to sexual and reproductive health services without weakening interventions for key populations. Condom distribution, pre‐exposure prophylaxis, partner testing, prevention of vertical transmission, and confidential counseling should be integrated into primary healthcare and maternal health services. Spouses and regular partners of migrants and people from key populations should be offered voluntary testing and prevention services without coercion or stigmatizing assumptions. Fourth, harm‐reduction programs remain essential. Opioid agonist therapy, needle‐and‐syringe programs, overdose prevention, HIV and hepatitis testing, and immediate ART initiation should remain central components of the national response. Surveillance systems should periodically validate registered transmission categories through confidential behavioral assessments. Where scientifically and ethically appropriate, molecular, phylogenetic, or network analyses may help identify transmission clusters that are not apparent from routine exposure classifications. Finally, declining mortality should not be interpreted as evidence that the epidemic is controlled. Sustained viral suppression, treatment monitoring, drug‐resistance surveillance, uninterrupted ART supplies, and long‐term retention remain necessary to preserve existing gains. Stronger integration of surveillance, clinical, migration, and behavioral data would enable more precise identification of populations experiencing delayed diagnosis or treatment interruption.

### 4.4 Strengths and Limitations

The principal strength of this study is the use of the standardized GBD 2021 framework, which enabled evaluation of national trends over more than three decades using comparable prevalence, mortality, and DALY measures. The study also examined age‐ and sex‐specific patterns and benchmarked Kyrgyzstan against Middle‐SDI and global reference estimates. Explicitly separating the observed 1990–2021 estimates from the 2022–2025 projections improved methodological transparency. Several limitations should be considered. First, GBD estimates are modeled and depend on the availability and quality of national surveillance, mortality, registry, and published data. Sparse or incomplete data may increase uncertainty, particularly for earlier years and narrowly defined demographic subgroups. Second, the 2022–2025 values are projections based on continuation of the 2010–2021 trend. They cannot account for future changes in migration, prevention funding, ART coverage, treatment interruption, surveillance practices, or national policy. Third, transmission mode is not a standard GBD outcome. The transmission findings were obtained from external surveillance sources and may be affected by non‐disclosure and misclassification. Fourth, the ecological design does not permit individual‐level causal conclusions regarding the effects of ART, migration, marital mobility, injecting drug use, or sexual behavior. Fifth, global and Middle‐SDI aggregates contain substantial between‐country heterogeneity and should be interpreted as contextual benchmarks rather than direct comparators. Finally, exact regression‐derived EAPCs and their 95% confidence intervals require the complete annual GBD series; annualized changes calculated from the 2010 and 2021 endpoints should not be interpreted as equivalent to formal EAPCs.

## 5 Conclusion

Kyrgyzstan has achieved meaningful reductions in HIV‐related mortality and DALY rates since approximately 2010, but HIV prevalence has continued to increase. Adults aged 20–49 years and males experienced the greatest burden, indicating the need for age‐ and sex‐responsive interventions. The increasing proportion of diagnoses registered as heterosexual exposure likely reflects a combination of genuine sexual transmission, migration, marital and partner mobility, behavioral change, and possible underreporting of stigmatized exposures. Further progress will require earlier diagnosis, sustained ART and viral suppression, expanded harm reduction, differentiated services for key populations, and voluntary outreach to migrants and their sexual partners. Strengthened behavioral surveillance and validation of registered transmission categories are also needed. Update the short‐term projections when a newer GBD estimation cycle or validated national data become available.

## Author Contributions

**Shafee Ur Rehman:** conceptualization, investigation, writing – original draft, writing – review and editing, visualization, validation, methodology, software, project administration, formal analysis, data curation, supervision, resources.

## Funding

The author has nothing to report.

## Ethics Statement

This study was based exclusively on secondary, aggregated, and anonymized data derived from publicly available sources within the Global Burden of Disease (GBD) framework and national surveillance summaries. No individual‐level or identifiable data were used. Therefore, ethical approval and informed consent were not required.

## Conflicts of Interest

The author declares no conflicts of interest.

## AI Use

Grammarly was used for grammar and language modification of the manuscript.

## Transparency Statement

The corresponding author, Shafee Ur Rehman, affirms that this manuscript provides an honest, accurate, and transparent account of the study conducted and reported. No important aspects of the study have been omitted, and any deviations from the original study plan have been appropriately disclosed and explained.

## Supporting information


Supporting File

## Data Availability

The data that support the findings of this study are available from the corresponding author upon reasonable request. All data used in this study are available from publicly accessible sources, including the Global Burden of Disease study and national HIV surveillance reports. The datasets analyzed during the current study are available from the corresponding author upon reasonable request.
