# Supplementary material for: HIV Burden Trends in Kyrgyzstan, 1990–2025: A National Analysis of GBD 2021 Estimates and Short‐Term Projections
Source: Health Sci Rep. 2026 Sep 22;9(10):e73279. doi: 10.1002/hsr2.73279 (PMC13598283; doi:10.1002/hsr2.73279)
Supplement: Supplementary file 1 [file HSR2-9-e73279-s001.docx]

**SUPPLEMENTARY MATERIALS**

**HIV Burden Trends in Kyrgyzstan, 1990–2025: A National Analysis of GBD 2021 Estimates and Short-Term Projections**

# Supplementary Note S1. Scope, data provenance, and analytical boundaries

The primary historical analysis covers 1990–2021 and is aligned with the Global Burden of Disease Study 2021 (GBD 2021). Values presented for 2022–2025 are author-derived short-term projections and are not official GBD estimates. The numerical tables below reproduce the anchor-year values used in the manuscript. National and UNAIDS surveillance information was used only to contextualize key-population sizes, programme coverage, and registered transmission categories; it was not statistically pooled with GBD estimates.

Transmission mode is not a standard GBD cause measure. Consequently, statements concerning heterosexual exposure, injecting drug use, male-to-male sexual exposure, and mother-to-child transmission should be interpreted as descriptive surveillance classifications. They may be affected by non-disclosure, stigma, and exposure misclassification.

# Supplementary Table S1. Data dictionary

| Variable | Unit | Operational definition | Provenance |
| --- | --- | --- | --- |
| People living with HIV | Count | Estimated number of prevalent HIV infections in a specified year | GBD 2021; projection after 2021 |
| ASPR | Rate per 100,000 | Age-standardized prevalence rate using the GBD standard population | GBD 2021; projection after 2021 |
| HIV-related deaths | Count | Deaths attributable to HIV/AIDS-related causes | GBD 2021; projection after 2021 |
| ASDR | Rate per 100,000 | Age-standardized HIV death rate | GBD 2021; projection after 2021 |
| DALYs | Count | Years of life lost plus years lived with disability | GBD 2021; projection after 2021 |
| Age-standardized DALY rate | Rate per 100,000 | DALYs standardized using the GBD standard population | GBD 2021; projection after 2021 |
| Annualized change | Percent per year | Geometric average annual change between two observed endpoints | Author calculation |
| Registered transmission mode | Percent of reported cases | Surveillance-assigned exposure category; not a GBD-modelled outcome | National/UNAIDS context |

# Supplementary Table S2. Numerical estimates used in the manuscript

| Indicator | 1990 | 2000 | 2010 | 2021 | 2025 projection* |
| --- | --- | --- | --- | --- | --- |
| People living with HIV, n | ~1,020 | ~3,450 | ~7,980 | ~12,430 | ~15,180 |
| ASPR per 100,000 | 4.1 | 11.6 | 18.9 | 21.7 | 22.8 |
| HIV-related deaths, n | ~120 | ~460 | ~680 | ~520 | ~470 |
| ASDR per 100,000 | 0.6 | 2.4 | 3.1 | 2.2 | 1.9 |
| Total DALYs, n | ~9,800 | ~38,400 | ~56,900 | ~49,200 | ~45,600 |
| Age-standardized DALY rate per 100,000 | 42.5 | 148.2 | 201.6 | 172.4 | 158.3 |

**The 2025 column contains author-derived short-term projections. Historical values through 2021 are the anchor estimates used in the manuscript. Approximate counts are identified with a tilde (~).*

# Supplementary Table S3. Annualized changes during 2010–2021

| Indicator | R2010 | R2021 | Annualized change, %/year | Direction |
| --- | --- | --- | --- | --- |
| People living with HIV | 7,980 | 12,430 | +4.11 | Increasing |
| ASPR | 18.9 | 21.7 | +1.26 | Increasing |
| HIV-related deaths | 680 | 520 | −2.40 | Decreasing |
| ASDR | 3.1 | 2.2 | −3.07 | Decreasing |
| Total DALYs | 56,900 | 49,200 | −1.31 | Decreasing |
| Age-standardized DALY rate | 201.6 | 172.4 | −1.41 | Decreasing |

*Annualized changes are endpoint-based geometric rates, not regression-derived EAPCs. They should not be accompanied by regression confidence intervals or P values.*

# Supplementary Note S2. Calculation and worked examples

The annualized percentage change between 2010 and 2021 was calculated as:

**Annualized change (%) = 100 × [(R₂₀₂₁/R₂₀₁₀)¹⁄¹¹ − 1]**

Worked example for the age-standardized prevalence rate:

100 × [(21.7/18.9)¹⁄¹¹ − 1] = +1.26% per year

Worked example for the age-standardized death rate:

100 × [(2.2/3.1)¹⁄¹¹ − 1] = −3.07% per year

Worked example for the age-standardized DALY rate:

100 × [(172.4/201.6)¹⁄¹¹ − 1] = −1.41% per year

A formal estimated annual percentage change (EAPC) requires the complete annual series. It is obtained by fitting ln(Ry) = α + βy + εy and calculating 100 × [exp(β) − 1]. Because the manuscript dataset available for this supplement contains selected anchor years, endpoint-based annualized changes were reported to avoid unsupported confidence intervals.

# Supplementary Table S4. Subgroup and transmission-mode interpretation

| Population/category | Summary finding | Evidence type | Interpretive caution |
| --- | --- | --- | --- |
| Adults aged 20–49 years | >65% of total HIV-attributable DALYs | Observed/projected burden summary | Priority age group; not an individual-level causal result |
| Males | Approximately 60–65% of DALYs | Sex-specific burden summary | Potential explanations require individual-level data |
| Females | Approximately 35–40% of DALYs | Sex-specific burden summary | Lower aggregate burden does not imply low risk in partners of key populations |
| Injecting-drug-use exposure | Predominant registered category in the earlier epidemic | Surveillance context | May be underreported |
| Heterosexual exposure | Increasing registered share in later years | Surveillance context | May reflect genuine spread, migration, marital networks, or over-assignment |
| Male-to-male sexual exposure | Smaller registered share | Surveillance context | May be affected by stigma and non-disclosure |
| Mother-to-child transmission | Small but persistent registered contribution | Surveillance context | GBD estimates do not identify the route of individual pediatric infections |

# Supplementary Table S5. Projection assumptions and limitations

| Component | Assumption | Implication |
| --- | --- | --- |
| Forecast horizon | 2022–2025 | Short horizon limits—but does not eliminate—forecast uncertainty |
| Trend basis | Continuation of the post-2010 trajectory | Assumes no structural break after 2021 |
| Functional form | Log-linear continuation | Assumes approximately constant proportional change |
| ART and testing | No explicit intervention covariates | Unexpected scale-up or disruption can alter outcomes |
| Migration | Not modelled explicitly | Labour mobility or displacement may change transmission and retention |
| Policy and financing | Held implicitly at recent-trend conditions | Funding shocks and policy changes are not anticipated |
| Surveillance practice | Assumed sufficiently comparable over time | Diagnostic or classification changes may alter recorded patterns |
| Interpretation | Scenario-based projection | Not an official GBD 2025 estimate |

# Supplementary Table S6. Figure provenance and interpretation

| Figure | Content | Data provenance | Required interpretation |
| --- | --- | --- | --- |
| Figure 1 | ASPR trend | GBD-aligned anchor estimates through 2021; author projection thereafter | Projected segment must be visually distinguished |
| Figure 2 | ASDR trend | GBD-aligned anchor estimates through 2021; author projection thereafter | Do not describe 2025 as observed |
| Figure 3 | Age-standardized DALY rate | GBD-aligned anchor estimates through 2021; author projection thereafter | Do not interpret overlapping uncertainty bands as hypothesis tests |
| Figure 4 | Age- and sex-specific DALY burden | Subgroup estimates/projections | Age- and sex-specific uncertainty should be retained |
| Figure 5 | Global and Middle-SDI benchmarking | Harmonized reference aggregates | Descriptive benchmarking; not causal comparison |
| Figure 6 | Registered transmission categories | National/UNAIDS surveillance context | Not a GBD-modelled transmission analysis |
| Figure 7 | Summary figure | Synthesis of manuscript results | Each panel should retain estimate/projection labels |

# Supplementary Note S3. Reproducibility code

The following R code reproduces the endpoint-based annualized changes reported in Supplementary Table S3:

\# Anchor-year dataset used in the manuscript

d <- data.frame(

indicator = c('PLHIV', 'ASPR', 'Deaths', 'ASDR', 'DALYs', 'ASR_DALY'),

value_2010 = c(7980, 18.9, 680, 3.1, 56900, 201.6),

value_2021 = c(12430, 21.7, 520, 2.2, 49200, 172.4)

)

\# Geometric average annual percentage change over 11 years

d$annualized_change <- 100 * ((d$value_2021 / d$value_2010)^(1/11) - 1)

round(d$annualized_change, 2)

\# Formal EAPC template for a complete annual dataset named annual_data

\# Required columns: year, indicator, rate

\# fit <- lm(log(rate) ~ year, data = subset(annual_data, indicator == 'ASPR'))

\# beta <- coef(fit)[['year']]

\# se <- summary(fit)$coefficients['year', 'Std. Error']

\# eapc <- 100 * (exp(beta) - 1)

\# ci <- 100 * (exp(beta + c(-1, 1) * 1.96 * se) - 1)

# Supplementary Note S4. Transparency and reproducibility statement

The corresponding author, Shafee Ur Rehman, affirms that the manuscript and these supplementary materials provide an honest, accurate, and transparent account of the study. Historical GBD-aligned estimates and author-derived projections have been explicitly distinguished. No regression-derived confidence intervals were assigned to endpoint-based annualized changes. Any future deposition of the complete annual GBD export should retain the original query settings, extraction date, uncertainty intervals, and analytical code.

Data availability: GBD 2021 estimates are accessible through the Global Health Data Exchange, subject to its terms of use. Contextual HIV programme and key-population indicators are available through UNAIDS and the cited national or regional reports.
